# Antibody levels to multiple malaria vaccine candidate antigens in relation to clinical malaria episodes in children in the Kasena-Nankana district of Northern Ghana

**DOI:** 10.1186/1475-2875-10-108

**Published:** 2011-05-01

**Authors:** Daniel Dodoo, Frank Atuguba, Samuel Bosomprah, Nana Akosua Ansah, Patrick Ansah, Helena Lamptey, Beverly Egyir, Abraham R Oduro, Ben Gyan, Abraham Hodgson, Kwadwo A Koram

**Affiliations:** 1 Department of Immunology, Noguchi Memorial Institute for Medical Research, University of Ghana, P.O. Box LG581, Accra, Ghana; 2 Navrongo Health Research Centre, P.O. Box 114, Navrongo, Ghana; 3 Department of Biostatistics, School of Public Health, University of Ghana, P.O. Box LG 13, Accra, Ghana; 4 Department of Epidemiology, Noguchi Memorial Institute for Medical Research, University of Ghana, P.O. Box LG581, Accra, Ghana

## Abstract

**Background:**

Considering the natural history of malaria of continued susceptibility to infection and episodes of illness that decline in frequency and severity over time, studies which attempt to relate immune response to protection must be longitudinal and have clearly specified definitions of immune status. Putative vaccines are expected to protect against infection, mild or severe disease or reduce transmission, but so far it has not been easy to clearly establish what constitutes protective immunity or how this develops naturally, especially among the affected target groups. The present study was done in under six year old children to identify malaria antigens which induce antibodies that correlate with protection from *Plasmodium falciparum *malaria.

**Methods:**

In this longitudinal study, the multiplex assay was used to measure IgG antibody levels to 10 malaria antigens (GLURP R0, GLURP R2, MSP3 FVO, AMA1 FVO, AMA1 LR32, AMA1 3D7, MSP1 3D7, MSP1 FVO, LSA-1and EBA175RII) in 325 children aged 1 to 6 years in the Kassena Nankana district of northern Ghana. The antigen specific antibody levels were then related to the risk of clinical malaria over the ensuing year using a negative binomial regression model.

**Results:**

IgG levels generally increased with age. The risk of clinical malaria decreased with increasing antibody levels. Except for FMPOII-LSA, (p = 0.05), higher IgG levels were associated with reduced risk of clinical malaria (defined as axillary temperature ≥37.5°C and parasitaemia of ≥5000 parasites/ul blood) in a univariate analysis, upon correcting for the confounding effect of age. However, in a combined multiple regression analysis, only IgG levels to MSP1-3D7 (Incidence rate ratio = 0.84, [95% C.I.= 0.73, 0.97, P = 0.02]) and AMA1 3D7 (IRR = 0.84 [95% C.I.= 0.74, 0.96, P = 0.01]) were associated with a reduced risk of clinical malaria over one year of morbidity surveillance.

**Conclusion:**

The data from this study support the view that a multivalent vaccine involving different antigens is most likely to be more effective than a monovalent one. Functional assays, like the parasite growth inhibition assay will be necessary to confirm if these associations reflect functional roles of antibodies to MSP1-3D7 and AMA1-3D7 in this population.

## Background

In malaria endemic regions, clinical malaria is responsible for high morbidity and mortality in less than five year old children and pregnant women. In these regions, individuals develop a partial 'non-sterile' immunity against erythrocytic stage disease in an age and exposure dependent manner and, therefore, older individuals suffer less clinical symptoms and disease complications. Sero-epidemiological studies show three sequential phases of development of acquired immunity to malaria: first, immunity to life-threatening disease; second, immunity to symptomatic infection; and only then, can the third phase, partial immunity to parasitization be achieved [1,2]. Passive transfer of antibodies from malaria-immune adults have been successfully used in the treatment of malaria patients [3,4], suggesting a crucial role of antibodies in immunity to malaria.

Several studies have reported associations between levels of antibody to various malaria parasite specific antigens and reduced risk of infection [5-9]. However, as yet, the precise antigenic targets of protective immunity to malaria remain largely unknown as findings from different correlates of antibody mediated immunity studies are often conflicting in their conclusions. Thus, there is presently no single immunological correlate of protection to clinical malaria, and those described do not sufficiently account for the overall variation in susceptibility observed in a population [10].

Several antigens due to their structures and locations have been deemed of importance in inducing protective antibodies against clinical malaria of the erythrocytic stage of the parasite. These include the merozoite surface proteins (MSP1, MSP2, MSP3, etc.) and the apical membrane antigen - 1 (AMA1), EBA-175 RII and GLURP [6,7,9,9,11-13], but the mechanism of action of these antibodies *in vivo *remains unclear [7].

In this longitudinal study, baseline IgG levels to ten malaria vaccine candidate antigens, namely, GLURP R0, GLURP R2, MSP3 FVO, AMA1 FVO, AMA1 LR32, AMA1 3D7, MSP1 3D7, MSP1 FVO, FMP011 (LSA-1) and EBA175RII were measured by the multiplex assay in plasma samples of 1 to 6 year old children, living in a malaria endemic region and the levels related to the risk of clinical malaria estimated over a one year period. The multiplex technique which has been validated and shown to have high correlation with the traditional ELISA technique in malaria antibody measurements and which has a higher detection range [14] was the preferred assay of choice for this study. In studies involving infants and children where only small volumes of samples are obtained and antibody measurements to multiple antigens are required as in this study, the traditional ELISA method is limited by the large sample volumes required. This study was aimed at elucidating which of the antibodies to the various antigens could act individually or in a concerted manner to confer immunity to malaria in the studied population.

## Methods

### Study site and population

The study was conducted in the Kassena-Nankana District (KND) of the Upper East region of northern Ghana. This is a savannah region where the people are mainly subsistence farmers. There are two main seasons, a dry season from about October to April and a wet season from approximately May to October. Malaria transmission occurs throughout the year with distinct patterns during the two seasons. The estimated malaria attack rate is approximately 3.5 attacks per child per year. The district is under the Navrongo Demographic Surveillance System (NDSS), which administers a complete population census of the population every 90 days. Details of the study area and population have been published elsewhere [15]. The KND is served by the Navrongo Health Research Centre, the Navrongo War Memorial Hospital and four other health centres. The study enrolled children between the ages from 1 to 6 years in KND, who planned to remain in the district for at least the entire duration of the study (one calendar year) and their parents or guardians consented to the study.

### The study design

The study enrolled 325 children between one and six years old independent of gender and with no regards to ethnicity. Children were randomly generated from the database of the NDSS and subsequently contacted in the recruitment exercise for enrolment. Only those whose parents/guardians agreed to be part of the study keeping all study required protocols were enrolled within the month of May 2004 (over a period of three weeks). These children were passively followed over one calendar year (May 2004 to May 2005) during which clinical, haematological and parasitological data were collected at the beginning of the study and every two months. Fingerprick blood samples (0.5-1.0 ml) were used to determine haemoglobin concentrations with a Hemocue Hb 201 (Angelhom, Sweden) and malaria infection. Giemsa-stained thin and thick malaria blood films (MBFs) were done and examined by two microscopists independently for parasites. An enhanced passive follow up approach was adopted where parents and guardians were encouraged to bring study participants to the health service facilities for any perceived illness. Field workers were also placed in the community to assist parents and guardians to access medical care. During every visit to the health facility, the participants were evaluated with a morbidity questionnaire and a physical examination by study clinicians. A blood smear was also taken and a rapid malaria diagnostic test (DiaMed Optimal Rapid Malaria test) done. Children suffering from uncomplicated malaria were treated with chloroquine and sulphadoxine-pyrimethamine (SP)(Fansidar^®^) as the first-line drugs according to Ghana Ministry of Health policy at the time. Those with severe forms of the disease were referred for free treatment with intravenous quinine at the Navrongo War Memorial Hospital. After discharge, the subjects were physically and clinically examined by a study physician and malaria smear and haemoglobin determination were made after which the subject was allowed to resume participation in the study. Other health conditions were managed as per the prevailing guidelines of Ghana Ministry of Health.

Parasite densities were calculated by enumerating the parasites against 200 WBC. The baseline blood samples were used to measure antibody levels to 10 malaria antigens (GLURP R0, GLURP R2, MSP3 FVO, AMA1 FVO, AMA1 LR32, AMA1 3D7, MSP1 3D7, MSP1 FVO, LSA-1and EBA175RII) using the multiplex assay. The immunological data was related to the risk of clinical malaria within the study period of one year.

### Recombinant antigens

The malaria antigens used in this study included a recombinant GLURP-R0 containing the conserved non-repeat N-terminal region, (amino acids 25-514), and GLURP-R2 (amino acids 705-1178) of the carboxy-terminal repeat region, all expressed in *Escherichia coli *[16] (supplied by Dr. Michael Theisen from State Serum Institute, Copenhagen). The recombinant C-terminal MSP1 42 protein of the FVO and 3D7 strains were expressed in *E. coli *[17]; and AMA-1 FVO (amino acids 25-545) [18], 3D7 and LR32 strains expressed in yeast *Pichia pastoris*, (all donated by Dr. Laura B. Martin from Malaria Vaccine Development Branch of NIAID, NIH). The EBA-175 RII expressed in yeast *P. pastoris*, containing amino acid residues 144 to 753, was provided by Division of Microbiology and Infectious Diseases, (DMID) NIAID, NIH under contract NO1-AI-05421. USA. The recombinant MSP-3 of the FVO strain was expressed in *E. coli*, (supplied by Dr. Richard Shimp from NIAID, NIH), and the LSA-1 expressed in *E. coli *was donated by Dr. David E. Lanar of the Division of Malaria Vaccine Development, Walter Reed Army Institute of Research, USA.

### Antibody measurements

Antigen specific IgG levels were meseasured against these antigens using the multiplex assay, a suspension array technique as described in a previous publication [14] with modifications as described below. Antigens were coupled to the microspheres using a modification of the protocol from Luminex Corp. Carboxylated microspheres or beads stock (Luminex Corp., Austin, TX) with different spectral addresses (101, 102, 103, 104, 105, 106, 107, 117, 118, 119 and 120) were centrifuged at 2,000 rpm for 5 min. The bead pellets were then dispersed by sonication and vortexing. A specified amount of the bead suspension was then centrifuged at 12000 rpm for 2 minutes and supernatant aspirated. The pellet was washed twice in 80 ul of activation buffer (0.1 M NaH_2_PO_4_, pH 6.2), and then activated with 10 ul of a 50 mg/ml solution of 1-ethyl 3-(3-dimethylaminopropyl) carbodiimide hydrochloride (EDC) and 10 ul of a 50-mg/ml solution of sulfo-*N*-hyroxysulfosuccinimide (Sulfo NHS). The microspheres were mixed gently and incubated at room temperature (RT) ranging from 25°C to 28°C, in the dark for 20 min. The activated microspheres were then washed twice with 250 ul of coupling buffer [0.05 M of 2-(*N*-morpholino) ethanesulfonic acid or MES, pH 5.0] and re-suspended in 100 ul of coupling buffer. To determine the optimal concentration of protein for coupling, 1.6, 4.0, 10, 25, and 62.5 μg/ml of recombinant proteins and BSA were assessed. Various concentrations of the recombinant malarial antigens such as GLURP R2 at (1.6 μg/ml); GLURP-R0, AMA1-FVO, MSP1-FVO, MSP1-3D7, LSA-1(all at 10 μg/ml), MSP3 FVO, AMA1 LR32, and EBA175RII (all at 25 μg/ml); AMA1 3D7 at 62.5 μg/ml were added to the microspheres. They were then mixed gently by vortexing, followed by 20 seconds ultrasonic sonication. The total volume was adjusted to 500 μl by adding the coupling buffer. The microspheres were incubated at RT in the dark for 2 hours with rotation, and then washed twice with 500 μl of storage buffer (phosphate-buffered saline [PBS], pH 7.2, containing 1% BSA, and 0.02% Tween 20, and 0.05% sodium azide). The coupled microspheres were re-suspended in 500 μl of blocking/storage buffer and stored at 4°C in the dark until ready for use.

### Multiplexed assay

Stock suspensions of antigen-coated microspheres were thoroughly re-suspended by vortexing and sonication before use. Filter plates (Multiscreen BV; Millipore, MA) were washed with 200 μl of washing buffer (PBS plus 0.05% Tween 20) and then washed with 200 μl of dilution buffer (PBS plus 1% BSA) using a vacuum manifold (Biorad Laboratories, CA, USA) to pre-wet the plates. Then, 25 μl of mixed microspheres of the antigens were distributed to microtiter wells (4,000 microspheres/well) and 50 μl of diluted plasma (1:50 and 1:2000 dilutions) was added. The plates were placed on a Microplate Shaker (Ika-schuttlar, MTS4 Labor-technik) and incubated at RT in the dark for 1 hr at 500 rpm. The plates were washed five times with 200 ul washing buffer. A total of 25 ul of 5 ug/ml (1:200) of goat anti-human immunoglobulin G (KPL 176-1006,) was added to each well, and plates were incubated for 1 hr in the dark at RT on a microplate shaker. After the incubation period, plates were washed five times with washing buffer, and then 50 ul of strepavidin phycoerythrin (1:800) was added and incubated for 30 mins at RT in the dark on a shaker. The reader (Boiplex system, Luminex Corp., Austin, TX) was programmed to read a minimum of 100 microspheres per bead region, and results were expressed as mean fluorescent intensity (MFI), which is directly proportional to antibody concentration[14]. For quality control of the assays, a pool of plasma obtained from the blood bank in Accra and predetermined to have high levels of IgG to multiple malaria antigens was included in each assay as positive control (PC). In addition, a pool of plasma from non-malaria exposed US volunteers was included as negative control (NC) in each assay. The antibody levels detected for the multiplex assay as mean fluorescent intensity (MFI) in this study varied with the different antigens used, ranging from a minimum of 25 MFI to 15, 736 MFI.

The study was reviewed and approved by the IRBs of both Noguchi Memorial Institute for Medical Research and the Navrongo Health Research Centre.

## Results

### Pattern of *Plasmodium falciparum *infection and clinical malaria in the study area

**Figure 1:**
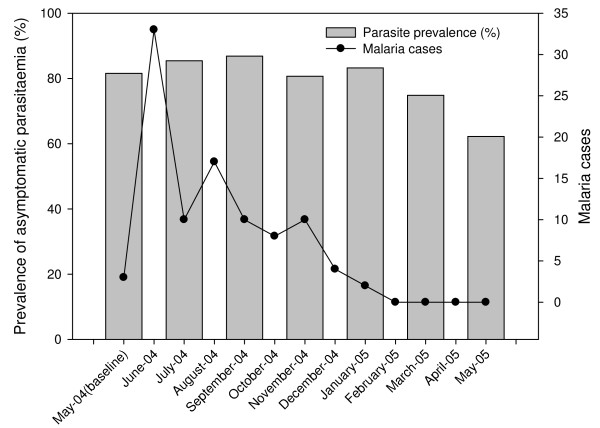
**Asymptomatic parasitaemia and clinical malaria episodes;** The prevalence of asymptomatic parasitaemia for each month is represented as a bar graph and the pattern of clinical malaria is shown as a line graph.

Of the total of 325 children, three hundred and fifteen had adequate samples for the immunological assays and the data generated from these was included in the analysis. Of this, 63 had at least one episode of clinical malaria during the one year period of the study and the incidence rate of clinical malaria decreased with age (Table 1). Asymptomatic *P. falciparum *infection was prevalent all year round; with about 60-80% study subjects carrying parasites throughout the study period (Figure 1). However, cases of clinical malaria peaked in June and dropped gradually to the lower levels typical of the low transmission season (Figure 1).

**Table 1:** Characteristics of the study population

Characteristics	Cumulative incidence of malaria	Child year at risk	No. of malaria episodes	Incidence rate per 100 child year (95% CI)
Age group				
12 <24 months	52.5% (21/40)	34.9	28	80 (55, 116)
24 <36 months	31.5% (17/54)	46.1	24	52 (35, 78)
36 <48 months	12.8% (10/78)	75.1	12	16 (9, 28)
48 <60 months	10.3% (8/78)	76.4	9	12 (6, 23)
60 <73 months	10.8% (7/65)	62.2	7	11 (5, 23)
				
Baseline *P. falciparum *parasitemia level				
Negative	41.4% (24/58)	52.1	31	59 (42, 84)
Positive	15.2% (39/257)	242.7	49	20 (15, 27)
				
TOTAL	20% (63/315)	295.1	80	27 (22, 34)

Clinical malaria is defined as history of fever or temperature>= 37.5 and parasite density>= 5000 u/l

### Association between antibody levels, age and clinical malaria

The sensitivity and specificity of defining clinical malaria cases may vary according to the intensity of malaraia transmission and the age of residents in malaria endemic areas [19-21]. Case definitions including parasiteamias of 2,500/μl blood or 5,000 parasites/ul blood in addition to reported or measured fever of ≥37.5°C have been used in previous reports [19,22], although for clinical practice a less strict case definition involving the history of fever is adopted. In this study involving children under seven years old in an area with sesonal, but intense malaria transmission, the case definition used was measured fever of ≥37.5°C in addition to parasitaemia at ≥5,000 parasites/μl and this was used to assess malaria specific antibody levels in relation to the risk of clinical malaria. Antibody levels in baseline samples tended to be lower in children who are negative for baseline parasitaemia and who are at higher risk to clinical malaria (Table 1). Children were considered to have a clinical malaria episode if they had axillary temperature of ≥37.5°C with a parasite density threshold greater than or equal to 5000/μl.

**Figure 2:**
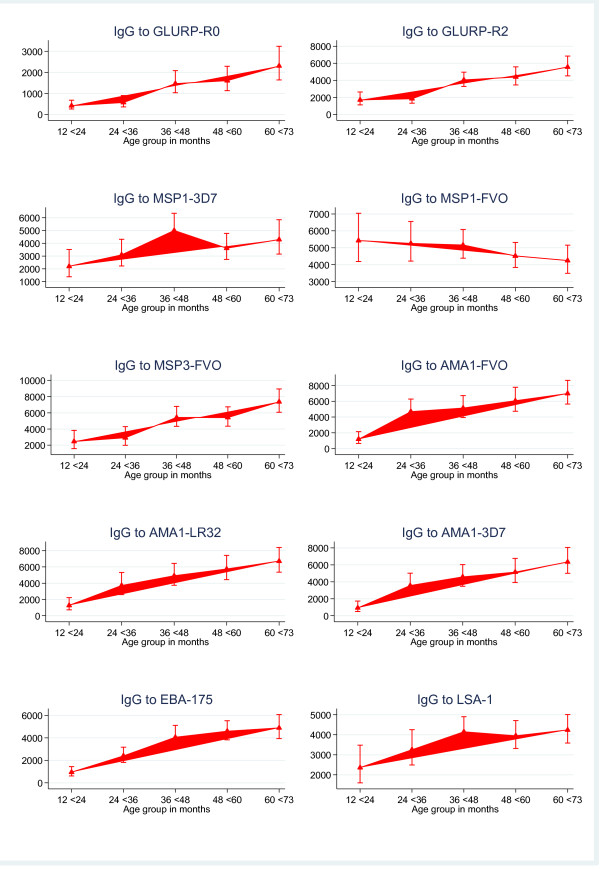
**Geometric mean antibody levels by age group**. Error bars show 95%CI.

Antibody titres to most of the antigens significantly increased with age (0.29≤r≤0.36, p < 0.0001, Figure 2), except for antibodies to LSA-1, which did not significantly correlate with age (r = 0.11, p = 0.051). After adjusting for the confounding effects of age, the antibody levels against all of the antigens tested, except LSA-1, were significantly associated with reduced risk of malaria (Table 2).

**Table 2:** Age-adjusted IRR for the association between total IgG level with clinical malaria

Antibody	Antigen	Crude IRR (95%CI)	IRR adjusted for age (95%CI)	P-value for adjusted IRR
IgG	GLURP-R0	0.76 (0.67, 0.86)	0.85 (0.75, 0.96)	0.01
	GLURP-R2	0.65 (0.55, 0.78)	0.77 (0.65, 0.91)	<0.01
	MSP1-3D7	0.72 (0.61, 0.85)	0.79 (0.69, 0.91)	<0.01
	MSP1-FVO	0.81 (0.63, 1.06)	0.73 (0.58, 0.93)	0.01
	MSP3-3D7	0.75 (0.63, 0.89)	0.86 (0.74, 1.01)	0.06
	AMA1-3D7	0.72 (0.63, 0.82)	0.80 (0.71, 0.91)	<0.01
	AMA1-FVO	0.72 (0.62, 0.83)	0.81 (0.71, 0.93)	<0.01
	AMA1-LR32	0.74 (0.64, 0.85)	0.85 (0.74, 0.96)	0.01
	EBA-175	0.66 (0.56, 0.78)	0.81 (0.68, 0.97)	0.02
	LSA-1	0.79 (0.64, 0.98)	0.89 (0.73, 1.09)	0.26

IRRs indicate the ratio of malaria incidence rates associated with a twofold increase in antibody level

In a final model, including age and all the immunological variables that showed evidence of reduced risk to clinical malaria in the univariate analysis, only MSP1-3D7 (IRR = 0.84 [95% CI, 0.73, 0.97, p = 0.02)] and AMA1-3D7 (IRR = 0.8 [95% CI, 0.74, 0.96, p = 0.01]) were shown to be independently associated with reduced risk of clinical malaria (Table 3).

**Table 3:** Adjusted incidence rate ratios for immunological variables independently associated with malaria risk in the final model

Immunological variables		Adjusted IRR (95%CI)	Wald P-value	LR test
MSP1-3D7 IgG	Values transformed to log base 2	0.84 (0.73, 0.97)	P = 0.02	
				
AMA1-3D7 IgG	Values transformed to log base 2	0.84 (0.74, 0.96)	P = 0.01	
				
Age group	12 <24 months	1		χ^2 ^= 21.24, p < 0.001
	24 <36 months	0.94 (0.44,2.01)	P = 0.88	
	36 <48 months	0.33 (0.14,0.77)	P = 0.01	
	48 <60 months	0.21 (0.08,0.51)	p < 0.01	
	60 <73 months	0.24 (0.09,0.66)	P = 0.01	

*IRR indicates the ratio of malaria incidence rates associated with a twofold increase in antibody level; *Parasite cut off is 5000+

## Discussion

The study found the persistence of asymptomatic *P. falciparum *parasitaemia in about 60 - 80% of the study population throughout the year. The incidence of clinical malaria however varied in parallel with the intensity of transmission and the onset of the rain as have been shown in previous studies in the same region [23]. The study area has been described as a malaria hyperendemic area with an entomological inoculation rate (EIR) of 418 infective bites and the age group used in this study was shown to have the highest prevalence of asymptomatic parasitaemia in an earlier study [24]. This could explain the persistence of asymptomatic parasitaemia year round.

The onset of rains increased vector population and might have allowed the introduction of novel parasites to which the children had no immunity from previous infection and, therefore, had the potential to cause more clinical disease [25]. Except for LSA-1 and MSP1-FVO, levels of IgG to all the antigens increased with age as shown in other studies [8,13]. Increasing IgG levels with age may reflect greater cumulative exposure but could also be due to older children having a more mature immune system [26]. The reason for the lack of correlation with age for anti-LSA-1 and MSP1-FVO antibodies, respectively, is unclear. It may be likely that LSA-1 is less immunogenic under the pattern of seasonal transmission in the study area in contrast to data from a study in a holoendemic region of Kenya, where infants were shown to be capable of mounting and sustaining strong anti-LSA-1 immune response in the first year of life [27], suggesting that levels of LSA antibodies might be more dependent on exposure rather than age. Interestingly, a study in Kenya [28] did not find any significant difference between antibody levels to LSA-1 in children (≤8 years old) and adults (≥ 18 years old) during both high and low malaria transmission seasons. The study showed a weaker correlation of IgG levels with age for MSP1-FVO compared to MSP1-3D7 implying that the 3D7 strain might be the predominant parasite in the study area but this will require parasite genotyping for confirmation. This finding is also very important as it underscores the relevance of strain specificity in design of vaccines. The relationship between malaria specific antibody response and risk of clinical malaria defined as reported or measured febrile temperature ≥37.5°C in the presence of parasitaemia ≥5,000 parasites/μl was examined, and nine of the 10 antigens showed significant association with reduced risk of clinical malaria whereas antibodies to LSA-1 did not, which was consistent with findings from other studies [13,26,29,30]. A correlation between LSA-1 antibody levels and rapid parasite clearance time [31] as well as protection in children [32] has been reported, but this could not be shown in other studies [33,34]. These differences could be due to several factors including transmission intensity and the age groups of the populations studied. Further studies might be necessary to establish the role of naturally acquired LSA-1 antibodies since it's been implicated in other studies to augment ADCI to enhance liver stage merozoites clearance [1,35].

In a final model involving all the immunological variables and age, IgG to MSP1-3D7 and AMA1-3D7 were found to be independently associated with reduced risk of clinical malaria indicating their potential as candidate antigens for vaccine development. However, these findings are only associations and the antibody responses to these antigens may be surrogate markers of other malaria antigen specific immunity that were not assessed in this study. The functionality of these antibodies that correlated with protection from clinical malaria in the present study can, however, be evaluated in a functional assay such as the *in vitro P. falciparum *growth inhibition assay (GIA) or the antibody-dependent cellular inhibition (ADCI) assay. Unlike other immuno-epidemiological studies which are usually conducted in older individuals whose immune response mechanisms may not be similar to that of children, the focus of this study was on young children whose immune systems may be in a state of flux as they develop partial immunity to malaria. This, therefore, gives us the opportunity to study more clearly which antibodies may be associated with a reduced risk to malaria.

## Conclusions

The results from this study also supports the view that a multivalent vaccine involving different antigens, such as MSP1-3D7 and AMA1-3D7, is most likely to be more effective than a monovalent one, at least in this area. These antigens can, therefore, be tested in the study site, which is under demographic surveillance and has high malaria transmission. This study also confirms the feasibility of using the multiplex assay to assess a panel of antigens with small volume of sample.

## Competing interests

The authors declare that they have no competing interests.

## Authors' contributions

DD designed the experiments and wrote the manuscript, FA performed and supervised field work, SB analysed the data, NAA supervised the field work, PA supervised the field work, HN and BE performed the experiments, ARO conducted and supervised the field work, BG designed the experiments, AH conceived the study, and KAK conceived the study and wrote manuscript. All authors read and approved the final manuscript.
